# Application of FT-NIR spectroscopy to the prediction of Chromium contamination in soil by evolutionary chemometrics

**DOI:** 10.1371/journal.pone.0341152

**Published:** 2026-01-27

**Authors:** Shaoyong Hong, Zhanhong Liang, Huazhou Chen, Jia Weng, Ken Cai, Xianchuan Wu

**Affiliations:** 1 School of Artificial Intelligence, Guangzhou Huashang College, Guangzhou, China; 2 School of Mathematics and Statistics, Guilin University of Technology, Guilin, China; 3 College of Automation, Zhongkai University of Agriculture and Engineering, Guangzhou, China; Beijing University of Chemical Technology, CHINA

## Abstract

Fourier-transform near-Infrared (FT-NIR) technology offers a promising alternative to traditional methods for detecting soil Chromium (Cr) contamination. However, the relationship between soil Cr content and the spectra may involve complex non-linear dynamics and data redundancy. Therefore, selecting spectral feature variables and constructing parametric scaling models for rapid estimation has become a focal point in current research. In this study, the parametric scaling support vector machine (PSSVM) method is proposed for optimizing the modeling parameters, the binary modified differential evolution (BDE) algorithm is designed for selecting the feature variables. In combination, a novel combined optimization system is established by embedding the PSSVM model into the BDE iterative process. The system (BDE-PSSVM) is validated by estimating the soil Cr content based on the FT-NIR spectral data. The soil samples are collected from the area around a centralized waste treatment base, serving as the research subject. The original spectral data underwent preprocessing using Savitzky-Golay smoothing. Subsequently, the samples were divided into the training and testing sets by the SPXY algorithm, where the testing samples are strictly excluded from the model training process. Feature selection and the parametric scaling model optimization are simultaneously performed by applying the BDE-PSSVM model. The most optimal model observes the minimal root mean square error of 8.114, which only carries 56 discrete variables. In comparison to some other counterpart modeling methods, the BDE-PSSVM uses less feature variables and yields the better prediction results. This finding indicates that the proposed BDE-PSSVM modeling system provides an efficient way for rapid estimation of soil Cr content in cooperation with the FT-NIR technology. The proposed system is expected to undergo testing for its application in detecting additional analytes.

## 1 Introduction

Heavy metal pollution in soil is mainly caused by various diverse human activities, such as industrial emissions, over use of pesticides and fertilizers and improper treatment of waste. It is worth noting that when the concentration of heavy metals exceeds the level that the environment elements can absorbed, it will cause harm to the ecological environment and human health [1]. In the ecosystem circulation, the heavy metals accumulate and is further transferred to parts of the plant or crops, and finally consumed by humans through the food chain in ecological cycle [2]. In sequence, the abnormal concentration of heavy metals conceived in soil can easily cause hemolysis and cell damage to humans, for even bringing serious irreversible hazards such as carcinogenesis and teratogenicity. Thus, rapid and accurate detection of heavy metal contents in soil is much crucial for soil remediation, restoration and reuse, in order to prevent harms to human health [3].

Typical methods for soil heavy metal detection mainly rely on reagent-involving chemical reactions, which are often destructive, time-consuming, and accompanied by the release of pollutants. Some existing traditional detection methods are originated from electrochemical techniques [4], but they are trapped intensive to the laboratory and not convenient for portable detection in a wide range large-scale performance [5]. Novel developments in spectral sensing technology have been verified for determining heavy metal contents. LIBS was the common spectroscopic technology applied for Cr detection. Combined with the studies on chemometrics, LIBS has been validated useful for rapid analysis of soil Cr [6–7]. Near-infrared (NIR) spectroscopy emerged for the detection of soil heavy metals. In common sense, the presence of heavy metal elements in soil has little spectral response in the NIR bands, which makes it difficult to quantify soil metals by NIR technology [8]. Fourier transform near-infrared (FT-NIR) spectroscopy is an analytical technique based on the interaction of the NIR light and the overtone and combination vibrations of substance molecules [9]. It correlates with the chemical composition and structure of substances to achieve qualitative identification or quantitative determination. This technique is non-destructive to samples, requires no complex pretreatment, and is applicable to samples in various forms such as solids, liquids, and gases. It features rapid analysis, with a single detection taking only a few minutes, and is environmentally friendly while reducing detection costs [10–11].

The change of heavy metals can be significantly revealed by using the diffused reflectance mode, and the FT-NIR bands exhibit its reasonable function to quantify the heavy metals in soil samples, when in highly support by the new advancements of chemoinformatic methods [12]. Previous publications reported that the main chemometric studies for NIR/FT-NIR detection of Cr is based on the classical PLS and PCA methods for modeling, accompanied with some variable selection technique [13]. Seldom work reports the study of machine learning or deep learning methods for Cr determination. The study on methodologies, especially on the AI and machine learning methods, has provided a new direction for the inversion of heavy metal contents [14]. Such that the NIR technology has become a powerful scientific tool for estimation of heavy metals in soil samples [15].

Method investigation to expose the FT-NIR information usually involves the reduction of noises, the selection of features, the optimization of models and the discussion of model stabilities [16]. When dealing with the multivariate spectral data, it is the core challenge to select appropriate spectral features (digitally revealed by variable selections for the spectral data). Although it is a common method for some studies to select relevant bands to benefit the model, the FT-NIR data, which goes integrated with signal amplification, still contain a lot of hidden information as stored in latent variables [17]. This is because there are strong correlations among spectral features, implying that the internal relationships in neighboring bands are often overlapped (Wang et al., 2020). With the increase of on-the-target bands, the curves of the spectra of a complex detected analyte (such as soil) cover each other, and gradually become blurred, unclear and confused, such that the colinearty can hardly be distinguished. This indicates that sample information extracted from the same waveband or from two partially repeated wavebands exhibit similar spectral properties, thereby increasing the difficulty for quantification, especially for the trace elements of heavy metals.

In dealing with the soil properties inherently included in the FT-NIR data, many chemometric methods have been studied, modified or even brand-new ones been proposed [18]. Waveband selection or variable selection is the traditional issue in spectral data analysis. Conventional methods involve interval screening, moving window and spatial transformation. Classical methods include principal component analysis, partial least squares and its variants (interval selection, moving window) [19]. Rapid estimation of heavy metal content in soil requires an excellent model to extract spectral information [20]. Many studies implied that the accuracy of nonlinear models is generally higher than that of linear models in estimating soil heavy metal content [21–22]. Chromium is a kind of trace content that needs a high-quality regression model to enhance the effect of model prediction. There is a model for estimating Chromium content was successfully constructed using surface soil samples. The study reported that extreme learning machine-based nonlinear model series are able to predict heavy metals to obtain high precision accuracy [23].

Nonlinear models require a lot of effort to refine their parameters. Support vector machine (SVM) is a good supervised machine learning method based on supporting vectors. It has been applied in NIR analysis of soil nutrients, food safety and medicines and pharmacies [24–25]. By adding support vectors, the correlation of the spectra be enhanced, potentially aiding in the rapid estimation of soil heavy metals [26]. SVM models works with evolutionary technique has been validated. An SVM model combined with GA was applied for the inversion calculation of heavy metal contents. The particle swarm optimization algorithm was employed to enhance the calibration model with support vectors. Through model refinement, a significant improvement in prediction accuracy is achieved [27]. These results demonstrate appreciating improvements in model accuracy. Attention is drawn that these studies used evolutionary method for optimizing the SVM parameters. Studies on evolutionary method for spectral feature selection are just at the beginning.

Suitable optimization algorithms can assist the nonlinear model to quickly find suitable parameters, thus improving the stability and prediction accuracy of the model for rapid estimation of soil heavy metals [28]. To address this issue, evolutional algorithms are preferred in recent years as they are engaged with iterative cycle of individual populations [29]. However, due to the fact that soil is a kind of complex analytes that contain diverse components, the spectral information extracted by the present algorithms may still lack relevant neighborhood attributes. Supporting vectors well address this issue. Actually, there are increasing evidences proving that evolutionary methods are able to select variables for spectral data analysis, such as the validated application by adaptively modification of GA [30] and the particle swarm optimization (PSO) [31]. These indicates that an evolutionary algorithm combined with AI-based modeling mode is able to support feature selection in spectral chemoinfometics, for the optimization of linear and nonlinear models. Differential evolution (DE) is a simple, easy-to-operate swarm intelligent algorithm, which provides an iterative optimization way for continuously refining the model, and synchronously‌ evaluating the prediction effects [32]. If DE method is appropriately designed for spectral feature selection, in fusion with the modeling parameter tuning process, it is prospectively effective to enhance the stability and predictive accuracy of the calibration models for rapid estimation of soil heavy metals.

This study focuses on training the SVM models for NIR spectral analysis of soil. The spectral data and the corresponding laboratory-tested Chromium (Cr) content are measured. This paper contributes in two aspects: (1) To propose the parametric scaling strategy for the optimization of the kernel supported SVM model for quantitative determination of the Cr contents by using the NIR spectral data; (2) To propose a binary-coding modified differential evolution (BDE) method to combine with the parametric scaling SVM (PSSVM) model; (3) To explore a deep optimization strategy in combined relation to both parameter tuning and feature selection. In quantitative comparison with the conventional PLS and SVM models, the proposed combined optimization strategy provides a novel robust chemometric methodology to improve the prediction accuracy of the NIR calibration model, which benefits the spectroscopic rapid determination of the Chromium content in soil samples. However, during the process of the proposed method applied to other dataset, it is still necessary to re-establish models for the different targeted samples. The key to the popularization and application of spectroscopic metrology models lies in passing the verification of modeling effectiveness.

## 2 Materials and preparations

### 2.1 Description of samples

Target soil samples were collected from around the Centralized Waste Treatment Base in north Zhongshan city of Guangdong province, China. The samples are mainly of the basal waterland soil and marginal red soil. A series of sampling sites are located. The distances between each adjacent site were slightly different, ranging about 3–5 meters. At each site, the five-point sampling method was applied in accordance with the national standard of China “*Soil Quality - Guidance on Sampling Techniques*” (GB/T 36197–2018). Centered at the designated point, a rectangular sampling area is demarcated. One sampling point is set at the center position of the rectangular area and at each of its four diagonal vertices, totaling 5 points. At each point, 10 cores were extracted from 0–20 cm in depth. Each core weighed about 20 grams. These cores were mixed together as the one-fifth part of a sample, then the 5-point sampling parts were mixed to comprise one sample (weighed approximately 1 kilogram). Each sample was separately placed in a labelled polythene plastic bag for storage, and convenient for delivery. The soil samples were firstly exposed to sunlight and proper ventilation for air-drying. Then, the they were screened and finely grounded to remove the clods, stones or twigs. Successively, the samples were filtered using a 10-mesh nylon sieve for particle refinement.

**Fig 1:**
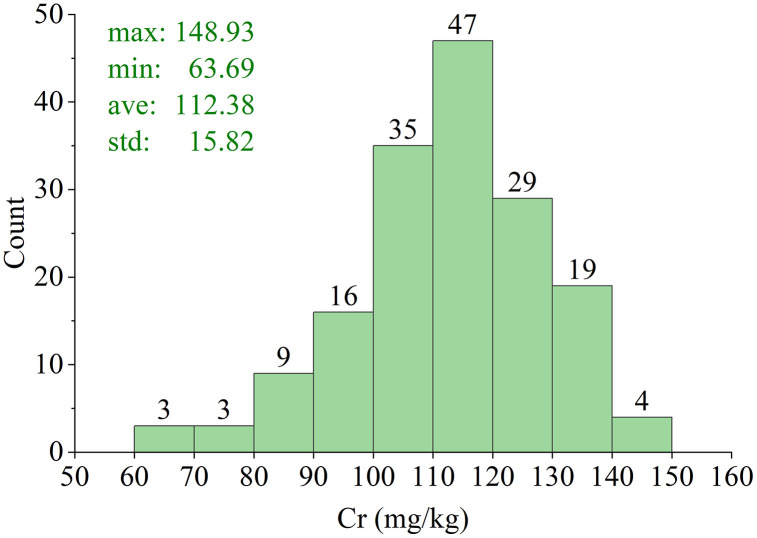
Histogram distribution of Cr content for the 165 soil samples.

Chromium is widely distributed in the copper smelting system, present in almost all intermediate products [33]. Chromium pollution has the most extensive impact, covering the majority of the surveyed land. Therefore, the measurement of Chromium is considered the primary focus of this study. Detailed analysis of Chromium (Cr) content in these soils was conducted. One hundred and sixty-five soil samples were utilized to measure the Cr content and NIR spectral data. The plastic-bag storage of each sample was divided into two parts. One part was used to collect the NIR spectral data, while the other part used to determine the Cr content obeying the conventional method regulated in the national standard of China “*Chromium metal—Determination of chromium content—The ammonium ferrous sulfate titrimetric method*” (GB/T 4702.1−2016). The histogram distribution of the Cr content is shown in Figure 1 for the 165 soil samples, and the basic statistics are also listed.

### 2.2 The FT-NIR measurement

The FT-NIR spectra of the 165 samples were one-by-one measured by using the BUCHI NIRMaster Spectrometer (BUCHI Labortechnik AG, Switzerland). The measurements were conducted in accordance with China’s national standards: GB/T 21186−2007 “Fourier Transform Infrared Spectrometers” and GB/T 6040−2019 “General Rules for Infrared Analysis”.

Each soil sample was placed in a petri dish, ensuring complete coverage of the bottom and surface to minimize background interference in data measurements. The entire sampling process is carried out in a dark environment to minimize the effects of external stray light and the data is collected via computer-link controls. During acquisition, the NIR optical path is to go perpendicularity to the soil observation surface, passe through the sample to near the bottom of the dish, and reflex back. Sensors and detectors are available to collect the reflectance signals. Then the signals were transformed and amplified by the FT section. During the spectroscopy measurement, the surrounding temperature and humidity were controlled consistent at 23 °C and 42%RH. Each sample was in queue to be automatically scanned for 64 times and the average data was recorded in the computer. The FT-NIR spectral range covered from 10000 to 4000 cm^-1^, with the digital resolution of 8 cm^-1^, thus to obtain the spectra containing a total of 1512 wavenumber points.

**Fig 2:**
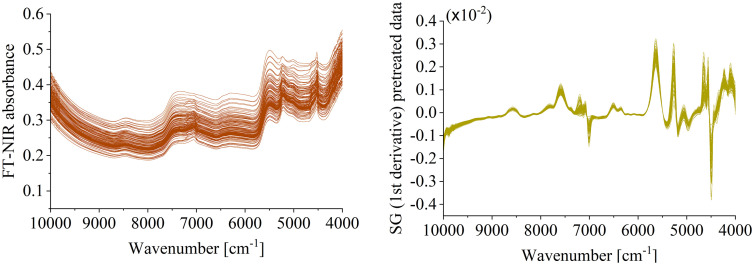
The FT-NIR spectra of 165 soil samples. **(a)** The raw data **(b)** The SG-pretreated data.

During the detection, sample properties, environmental and instrument-related factors are all potential to influence the quality of data, resulting in extraneous information such as noise, spikes, instrument-generated noise and stray light [34]. These interferences possibly increase the difficulties to find the valuable information and spectral features from the raw data. To address these issues, Savitzky-Golay (SG) filtering algorithm was used to smooth the data. This method is able to effectively reduce the impact of instrument background and drift on the signal. It is easy to obtain the pretreated spectral data which is optimally pretreated by the 1^st^ derivative performance of a cubic polynomial accompanied with a 37-point window. The raw spectra and the SG-pretreated curve were shown in Figure 2.

### 2.3 Sample set division for data training and testing

When the data is used for FT-NIR analysis, it is usually required to divide the sample pool into the sets for model training and testing. The testing sample set is not relevant to the model training process, while the training sample set is used to establish and the model, to tune the parameters/hyperparameters and to determine a suitable optimization strategy. In our experiment, we divide the 165 soil samples into the training set and the testing set with the ratio of 7:3, and the Sample Partitioning method based on joint X-Y distance (SPXY) [35] is applied. This division results in 115 samples allocated for training and 50 samples for testing. Descriptive statistical analysis was performed on the Cr contents for the training set and for the testing set (See Table 1). During the training process, the model is calibrated by using the 5-fold cross validation.

**Table 1:** Descriptive statistics of Cr contents for the training and testing sample sets, respectively.

(Unit: mg/kg)	No. of samples	Maximum	Minimum	Average	Standard Deviation
Training set	115	148.93	63.69	114.06	15.67
Testing set	50	143.13	67.07	108.51	15.61

As frequently used in spectral analysis, the two indicators of the root mean square error (RMSE), the Pearson correlation coefficient (R) and the coefficient of determination (R^2^) are employed for model evaluation [36]. They are formulated as:


$$\text{RMSE}=\sqrt{\frac{\text{1}}{n}\sum {\mathrm{\backslash nolimits}}_{i=\text{1}}^{n}{\left(y_{i}-{\tilde{y}}_{i}\right)}^{\text{2}}},$$ (1)

$$\text{R}=\frac{\sum_{i=\text{1}}^{n}\left(y_{i}-y_{m}\right)\left({\tilde{y}}_{i}-{\tilde{y}}_{m}\right)}{\sqrt{\sum_{i=\text{1}}^{n}{\left(y_{i}-y_{m}\right)}^{\text{2}}}\sqrt{\sum_{i=\text{1}}^{n}{\left({\tilde{y}}_{i}-{\tilde{y}}_{m}\right)}^{\text{2}}}},$$ (2)

$${\text{R}}^{\text{2}}=\text{1}-\frac{\sum_{i=\text{1}}^{n}{\left(y_{i}-{\tilde{y}}_{i}\right)}^{\text{2}}}{\sum_{i=\text{1}}^{n}{\left(y_{i}-y_{m}\right)}^{\text{2}}},$$ (3)

where, $y_{i}$ represents the Cr content of the i-th sample, ${\tilde{y}}_{i}$ is the FT-NIR modeling prediction value of the Cr content of the i-th sample; $y_{m}$ and ${\tilde{y}}_{m}$ are the mean values of the sets of $\left\{y_{i}|i=\text{1},\text{2}\ldots n\right\}$ and $\left\{{\tilde{y}}_{i}|i=\text{1},\text{2}\ldots n\right\}$, respectively; and n represents the numbers of participating samples in the targeted sample set. For convenient discussions, we denote RMSE, R and ${\text{R}}^{\text{2}}$ with the subscript CV for cross validation and the subscript T for testing.

## 3 Methodologies

Our research focused on machine learning methods for chemometric improvement in FT-NIR data analytical field, only involving non-destructive FT-NIR spectral measurements and surface soil scraping sampling, which would not cause any disturbance to the soil structure or the surrounding vegetation. Also, the centralized waste treatment base where the samples were collected is not a restricted or protected ecological zone, or a cultural relic site, or a private property. Thus, this is a kind of non-destructive and low-impact research activities in public non-protected areas. No permits were required.

### 3.1 Parametric scaling design for support vector machine

Support Vector Machine (SVM) is a commonly used stoichiometric method to establish a non-linear calibration model to minimize the structural risk. It uses a kernel function to map the variables from a low-dimensional space to a high-dimensional feature space, and establish the optimal decision function [37]. The aim of SVM optimization is to identify the following functional relationship


y=ω·φ(x)+b, (4)

where y is the targeted analyte (the Cr content of soil) and x (the NIR spectral data). Concerning on the minimization of structural risk, the constrain is defined in relation with the model coefficients and the prediction errors, i.e.,


$$\text{min}\;Q(\omega ,\varepsilon )=\frac{\text{1}}{\text{2}}\omega^{T}\omega +\frac{\text{1}}{\text{2}}\gamma \sum {\mathrm{\backslash nolimits}}_{i=\text{1}}^{n}\varepsilon_{i}^{\text{2}},$$ (5)

where ω and b are the coefficient for regression, γ is a tunable parameter for model regularization on avoiding overfitting; ε represents the error between y and x, which is denoted as $\varepsilon_{i}$ for each of the samples (i=1,2…n); and n is the number of participating samples.

The radial basis function (RBF) is frequently used as the kernel for specified mapping, as it can simplify the computation, and is endorsed stable and robust [38]. The RBF kernel is defined as using an exponential transform to illustrate the projection of x on anyone of the other variables $\left\{x_{i}|i=\text{1},\text{2}\ldots p\right\}$, namely,


$$\varphi (x)=\varphi \left(x,x_{j}\right)=\text{exp}\left(-\frac{{\left\|x-x_{i}\right\|}^{\text{2}}}{\sigma^{\text{2}}}\right),$$ (6)

where, σ represents the radial width of the RBF kernel function, which controls the nonlinear degree of the mapping.

The parametric scaling SVM (PSSVM) model is operated by collaborative tuning of γ and σ, which mainly works on the control of the optimal structure with minimum risk of falling into local optimization. By proposing the greedy grid search testing on the combined parameters (γ,σ), the SVM model can be precisely optimized [39].

However, even though that the PSSVM optimization works on greedy grid search, it does not provide an effective way for deep selection of informative variables. Feature selections in advance of modeling could have prospective improvement for model training and testing [40].

### 3.2 The algorithmic flow of binary differential evolution

Differential evolution (DE) algorithm is an iterative optimization technique based on swarm intelligence, which will launch continuous evolutionary updating evolutionary populations, and the difference among populations are evaluated [41]. Taking a set of variable combination as the population in the DE procedure, then the generation evolution of the population indicates the continuous updating of the selected informative variable combination.

According to the traditional DE method, the parameters include population size (NP), number of variables in the interval (L), crossover threshold (CT) and the maximal iteration (G). The binary-modified DE (BDE) method inherits the basic steps of DE, including the population initialization and the multi-round iteration of mutation, crossover and selection [42]. For improvement, BDE adopts 0–1 binary coding to decide whether the individual variables are selected, where Code 1 indicates that the variable is selected, while Code 0 means that the variable is not selected. This alternative operation simplifies the population evolution procedure. The main task is merely to test each individual variable if it adopts the changes of 0–1 binary coding during the mutation and crossover steps. Specifically, the BDE iterative optimization process can be designed in steps,

*Population initialization.* A population is taken as the solution to the modeling search of feature variables. The population is initialized and evolute in the increasing series of generations (g=0,1,2…G). Supposing the population is composed of NP individual variables, $\left\{V_{\text{1}},V\ldots V_{NP}\right\}$, coupled with NP 0–1 binary labels $\left\{s_{\text{1}},s_{\text{2}}\ldots s_{NP}\right\}$. Each individual label $s_{i}$ is created as an L dimension vector denoted as $s_{i}=\left\{s_{i}(q)|q=\text{1},\text{2}\ldots L\right\}$ where the q-th element adopts the binomial coding value of $s_{i}(q)=\text{1}\;\text{or}\;\text{0}$, which determines whether the corresponding individual $V_{i}$ is selected to the current generation (g) or not. Then the individuals are optimized in iterative cycles of mutation, crossover and selection. A fitness function is designed to evaluate the BDE evolutionary results for feature variable selection [43].

*Mutation.* The mutation of $V_{i}$ strictly follows the mutation of $s_{i}$. How an individual mutates is influenced by the other individuals (represented by the labels). Taking two other individuals as the influencing factors, the elements of $s_{i}$ is updated by calculation as follows,


$$\alpha_{i}^{g+\text{1}}(q)=s_{i}^{g}(q)+(-\text{1}{)}^{s_{i}^{g}(q)}\cdot \left|s_{\tau_{\text{1}}}^{g}(q)-s_{\tau_{\text{2}}}^{g}(q)\right|,\;\text{for}\;i=\text{1},\text{2}\ldots NP\;\text{and}\;q=\text{1},\text{2}\ldots L$$ (7)

where g points to the current generation and g+1 means the next generation; $s_{\tau_{\text{1}}}$ and $s_{\tau_{\text{2}}}$ represent the labels of any other two individuals which are randomly selected ($\tau_{\text{1}}\neq \tau_{\text{2}}\neq i$); $\alpha_{i}^{g+\text{1}}$ represent a candidate selection of $s_{i}^{g+\text{1}}$ by mutation. Equation (7) tells that whether the value at the q-th element of $s_{i}$ mutates depends on the mutated differences of the q-th of $s_{\tau_{\text{1}}}$ and $s_{\tau_{\text{2}}}$ (denoted as $d_{\tau }^{g}≜\left|s_{\tau_{\text{1}}}^{g}(q)-s_{\tau_{\text{2}}}^{g}(q)\right|$). If $\;d_{\tau }^{g}=\text{1}$, then $\alpha_{i}^{g+\text{1}}(q)$ will become the opposite side of $s_{i}^{g}(q)$ (changed from 0 to 1 or from 1 to 0); if $d_{\tau }^{g}=\text{0}$, then $\alpha_{i}^{g+\text{1}}(q)$ will be the same as $s_{i}^{g}(q)$. In this way, all elements of $s_{i}$ will be checked and refreshed from the g-th to become the candidate $\alpha_{i}^{g+\text{1}}$ at the (g+1)-th generation, when q goes through the L dimensions.

*Crossover.* Crossover takes place on the basis of the mutated individuals, with accurate correspondence with the label $\alpha_{i}^{g+\text{1}}$. The resultant crossover candidate individual is labeled as $\beta_{i}^{g+\text{1}}$. The calculation is monitored with a preset threshold (CT), and an instant crossover probability (CP) is produced for making the decision. If CP≤CT, then $\beta_{i}^{g+\text{1}}=\alpha_{i}^{g+\text{1}}$; otherwise, crossover is not accepted, and $\beta_{i}^{g+\text{1}}$ remains the same as $s_{i}^{g}$. In sequence, all individuals $\left\{V_{i}\right\}$ of the population will be updated according to the labels $\left\{\beta_{i}^{g+\text{1}}|i=\text{1},\text{2}\ldots NP\right\}$.

*Selection.* The fitness value is computed for selecting the evolved individuals at the (g+1)-th generation. The selection decision depends on the comparison of the candidate individuals labeled with $\left\{\beta_{i}^{g+\text{1}}\right\}$ to the original individuals labeled with $\left\{s_{i}^{g}\right\}$. If the fitness value is optimized, the individuals of the (g+1)-th generation (confirmedly denoted with labels $s_{i}^{g+\text{1}}$) is identified with the $\beta_{i}^{g+\text{1}}$ labels; otherwise, if not optimized, which means that this round of BDE evolution does not improve the set of feature variables, thus $s_{i}^{g+\text{1}}$ will inherit the original individuals of the g-th generation, namely,


$$s_{i}^{g+\text{1}}=\left\{ \begin{array}{cc} \beta_{i}^{g+\text{1}}, & \text{fitness}\;\text{optimized},\\ s_{i}^{g}, & \text{otherwise}. \end{array}\right.\;$$ (8)

Accordingly, the labels $\left\{s_{i}^{g+\text{1}}\right\}$ point to a refreshed set of improved feature variables $\left\{V_{i}^{g+\text{1}}\right\}$ by one round of BDE iteration. Along with the iteration goes to the end, the set of informative variables is well optimized, to enhance the model prediction performance.

### 3.3 The combined modeling framework of BDE-PSSVM

**Fig 3:**
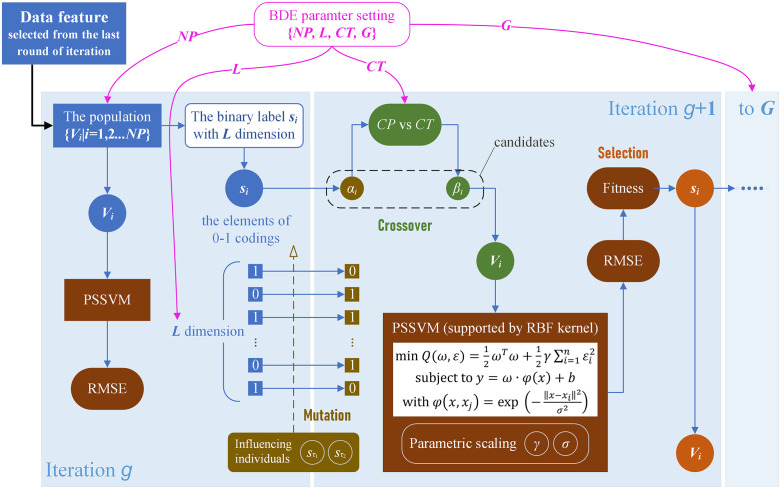
The flowchart of BDE-PSSVM modeling procedure.

In this study, we proposed the novel optimization strategy of combining the PSSVM model with the iterative BDE method. The combined modeling procedure performs along the BDE multi-round cycling calculation of mutation, crossover and selection, where PSSVM model is embedded into each step. The model predictive RMSE value is used to evaluate the immediate evolutionary fitness values. The flowchart of BDE-PSSVM modeling procedure is showed in Fig 3.

In experiment, the RBF supported SVM model is trained and tested with its combined parameters (γ,σ) being scaled as the values of $\left({\text{2}}^{i},{\text{2}}^{j}\right)$ for the tuning of i,j=1,2…10. The BDE iteration is tested with expansion on parameter tuning, for NP=1,2…8 (the size of population changes from 1 to 8), L={10,20…100} (any one population contains the number of individuals changing from 10 by step 10–100) and G=200 (for a maximum of 200 iterative rounds). The crossover is tested at four different thresholds of CT={0.2,0.4,0.6,0.8}. The fitness value is evaluated using the predictive RMSE value from the kernel supported SVM model. In this way, the NIR calibration model is refinedly optimized by the BDE-PSSVM computing methodology to obtain stable quantification results with prospective high prediction accuracy.

## 4. Results and discussion

### 4.1. SVM parameter scaling optimization

The PSSVM model was applied for the FT-NIR spectroscopic analysis to predict the soil chromium content, and the RBF function was used as the kernel. The regularization factor γ and the radial width σ of the RBF kernel had great influence on the prediction effect of the model. The parametric scaling tuning of the parameter group of (γ,σ) optimize the model and determine the Cr content of soil. The calibration samples were used to train the model, and (γ,σ) are adjustable during the training.

**Fig 4:**
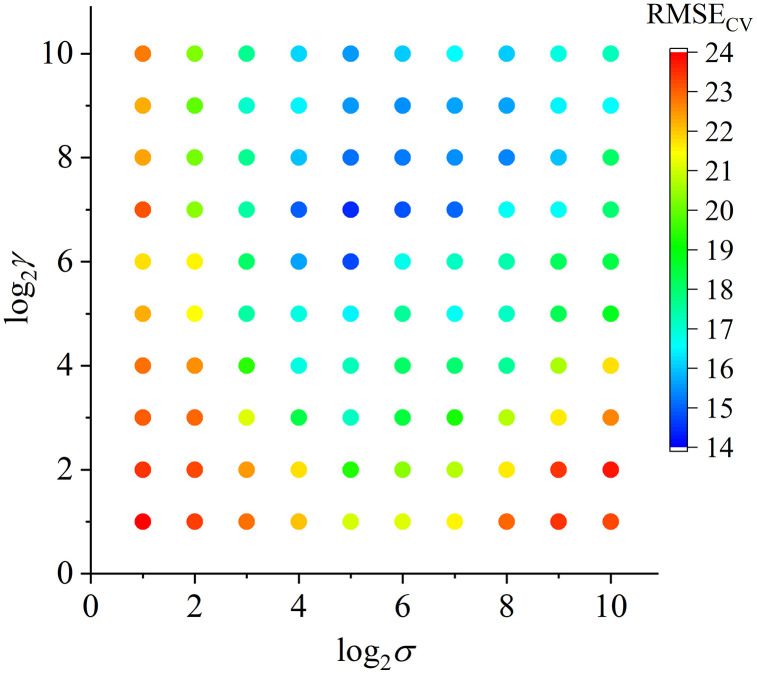
The grid search prediction results by parametric scaling of {γ,σ} for SVM model.

The parameters γ and σ were constructed as for grid search. The candidate values are preset as an integer exponential ${\text{2}}^{i}$ for screening i=1,2…10; thus, a total of 100 candidate combination of (γ,σ) were monitored. Each valuing for the combination of (γ,σ) determined one SVM model for NIR prediction. The trials are touching slowly from the initial prediction to the optimal result. By cross validation, the objective function value ${\text{RMSE}}_{\text{CV}}$ was calculated for training. In the way of parametric scaling, the ${\text{RMSE}}_{\text{CV}}$ conducted from each value of (γ,σ) are found, and we draw the scatter plot for internal comparison (see Fig 4). In the figure, we use the logarithm form to present the values of γ and σ, thus it is easily observed that the most optimal SVM model is constructed with ${\text{log}}_{\text{2}}\gamma =\text{7}$ and ${\text{log}}_{\text{2}}\sigma =\text{5}$, which deduce the optimized ${\text{RMSE}}_{\text{CV}}$ equaling to 14.48 mg/kg. Other than the optimal model, the advantage of parametric scaling is that it is able to simultaneously provide many different possible models with acceptable prediction results, such as the cases when ${\text{log}}_{\text{2}}\left(\gamma ,\sigma \right)$ equals to {(6,5),(7,4),(7,6),(7,7),(8,5),(8,6),(8,7),(9,5),(9,6),(9,7)}. These appreciating selections allow most possible choices for actual application.

However, the PSSVM model is established and trained based on the full-range spectroscopic data. The candidate combinations of parameters inevitably overlap the optimal results then obtain a not-so-better result through the kernel calculation, even though the model plays in a grid search scaling mode. To address this issue, differential evolutionary method is applied to select feature variables for optimizing the PSSVM model.

### 4.2. Feature selection by BDE-PSSVM optimization

**Fig 5:**
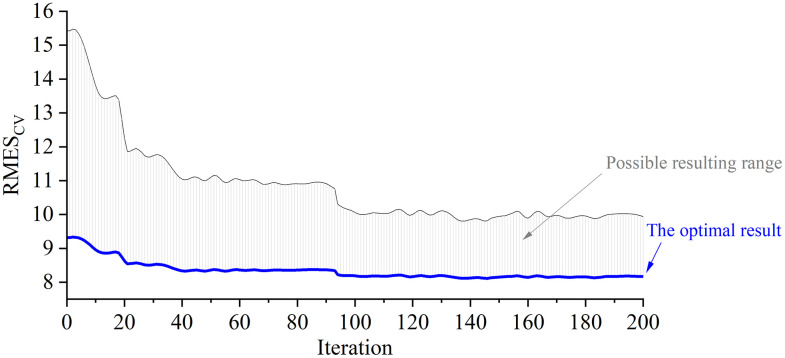
The iterative trend of BDE optimization in combination with PSSVM parametric scaling.

The model is beneficial by the combination of BDE-PSSVM optimization, in which BDE is functional to select informative features. The characteristics of the model is to ensemble the PSSVM modeling section into the BDE iterative optimizational process. The parameter expansion for BDE algorithm (i.e., the size of the population, the decomposed length of an individual, the increase of iteration times) can help enlarge the coverage of model training, and speed up the converge to the optimal solution. The initial 0–1 valuing of each individual is partially random but obeying the BDE formulations. The parameters {NP,L,CT,G} as preset in our experiment are tested for each of the BDE iterative rounds of mutation, crossover and selection. For the total 200 times of iteration, all possible PSSVM models are tested and the most optimal one is specially focused. In order to fully consider the influence of different combinations of the PSSVM modeling parameters, we highlight the most optimal modeling result at each step of BDE iteration (see Fig 5). By comparison on BDE iteration, the best BDE-PSSVM model is observed at the 146^th^ iteration. Fortunately, we can see the more applicable results that the model prediction results get stable close to the best model after around the 95^th^ iteration.

**Fig 6:**
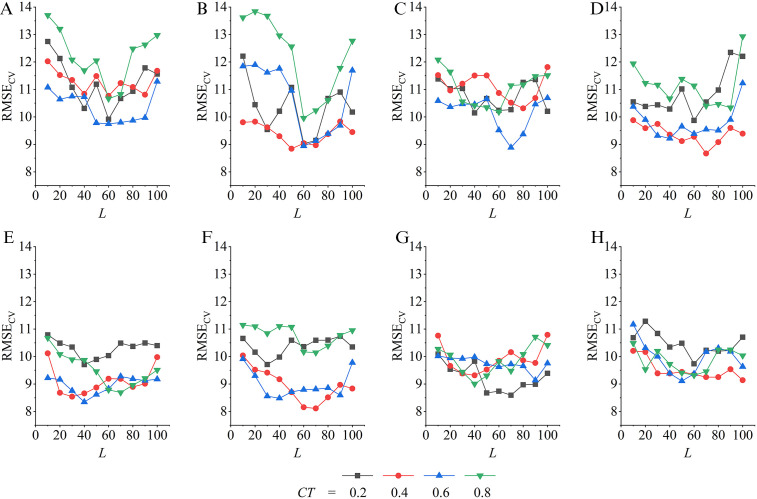
The BDE iterative results by tuning the specific parameters of {*NP, L, CT*}. **(a)** NP = 1 **(b)** NP = 2 **(c)** NP = 3 **(d)** NP = 4 **(e)** NP = 5 **(f)** NP = 6 **(g)** NP = 7 **(h)** NP = 8.

**Fig 7:**
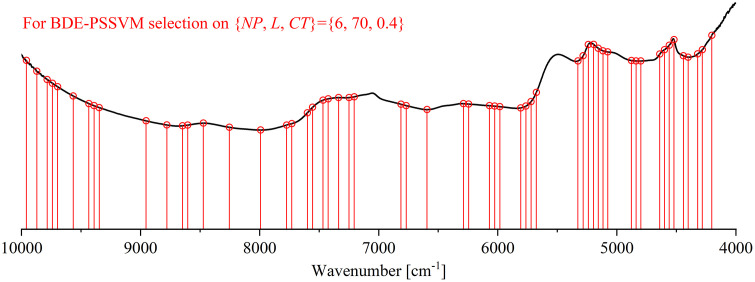
The 56 feature variables selected by BDE-PSSVM.

Combinations of informative variables were selected by the BDE-PSSVM model, which can be categorized into wavelength-based variable selection methods. The insight view of how the BDE parameter is tuned can help trigger the strategy for applicable feature selection. Other than the iteration times, the BDE parameters NP,L and CT are also monitored. For each combination of {NP,L,CT} when NP was tuned on {1,2…8}, L on {10,20…100} and CT on {0.2,0.4,0.6,0.8}, respectively, a total of 320 parametric BDE patterns are trained for 200 iterations, in which the PSSVM models are optimized in duration. The best refined models are observed for each pattern (see Fig 6). Every pattern determines one variable combination that points to a set of informative features. For example, the most optimal pattern is found on {NP,L,CT}={6,70,0.4}, it observes the minimal ${\text{RMSE}}_{\text{CV}}$ of 8.114, resulting in a combination of 56 variables that are most informative (See Fig 7).

### 4.3. Model verification and comparison

The feature variables trained by BDE-PSSVM model were selected as the informative variable combination for the optimal prediction of soil Cr content. Subsequently, the model with its optimal parameters and the selected feature variables are verified based on the testing samples, which is totally independent from the model training process.

To investigate the modeling efficiency, the BDE-PSSVM model is compared to some models combined with other evolutionary methods (GA and PSO) and some classical variable selection models (MWPLS, SiPLS). The model training results and testing results are showed in Table 2, the used number of variables is also listed in. Upon comparing with Table 2, it is evident that the BDE-PSSVM model exhibits the lowest RMSE value and the highest R value, while the applied number of feature variables are the least, which indicated that the regression model is simple. Contrast to the competitor models, the BDE-PSSVM achieves significant improvements in both model prediction accuracy and feature selection.

**Table 2:** Comparison of the optimal models by different methods with parametric scaling.

	No. of variables	${RMSE}_{CV}$	${𝐑}_{CV}$	${𝐑}_{CV}^{2}$	${RMSE}_{𝐓}$	${𝐑}_{𝐓}$
PSSVM	1512	14.485	0.882	0.808	16.274	0.873
BDE-PSSVM	56	8.114	0.931	0.864	10.639	0.908
GA-PSSVM	147	10.199	0.908	0.819	12.010	0.895
PSO-PSSVM	131	11.510	0.916	0.816	13.111	0.887
PLS	1512	17.730	0.859	0.790	19.716	0.847
MWPLS	220	15.453	0.877	0.759	17.641	0.863
SiPLS	192	13.772	0.891	0.796	17.792	0.878

The outputs of the proposed model were further compared with published results from recent years. For example, Wang et al. reported an ${\text{R}}_{\text{CV}}^{\text{2}}$ of 0.737 and an RPD of 3.000 for soil Cr prediction using the MEA-BP neural network model [44]. Han et al. established an SVM model, achieving a predictive RMSE of 2.20 and an R² of 0.77 [45]. Shirley et al. employed a PLS model coupled with MSC preprocessing, yielding a prediction RMSE of 7.57 and an R² of 0.76 [46]. Yuan et al. proposed a wavelength phase-out PLS model combined with repetition rate priority combination methods to improve soil Cr prediction via NIR spectroscopy, achieving an RMSE of 9.15 and an R of 0.843 [47]. In direct comparison with these previously reported NIR/FT-NIR modeling results, the proposed BDE-PSSVM model yielded an RMSE_CV_ of 8.114 and an R_CV_ of 0.931. The performance is superior to most of the previous studies. Moreover, the observed ${\text{R}}_{\text{CV}}^{\text{2}}$ of 0.864 indicates that the model has high explanatory power for soil Cr content, thereby ensuring optimal testing results.

## 5 Conclusions

To accurately predict the soil Cr content by FT-NIR spectroscopy, this study proposes a modified model optimization system based on BDE iterations in combination with the PSSVM modeling. The combined optimization model is functional to perform a deep learning strategy for grid search parameter tuning and for feature selection.

Based on the analysis by PSSVM model, a grid search parametric optimization way is proposed. Except observing the most optimal PSSVM model with parameters (γ,σ) equaling to $\left({\text{2}}^{\text{7}},{\text{2}}^{\text{5}}\right)$, we also provided a number of appreciating parameter combinations for model optimization, which are able to support real-scene applications. Moreover, the BDE-PSSVM modeling system is established based on the iteration of generations by modifying the DE method. The parametric scaling SVM modeling section is embedded in each round of BDE iteration, for adjusting the most optimal modeling parameters in steps. The BDE plays the 0–1 valuing role to select feature variables for model training and testing. Finally, the BDE-PSSVM model successfully select a set of 56 feature variables (see Fig 7) that are most informative for the quantitative prediction of soil Cr content. The model is able to well optimize the FT-NIR calibration model and improve the prediction effect, in comparison to the counterpart models, including some evolutionary algorithm of similar type, some classical linear and nonlinear modeling methods (SVM, PLS) and their variants for feature selection (see Table 2).

The research findings demonstrate that the BDE-PSSVM model performs remarkably well in modeling within the FT-NIR data. This discovery also reveals that, in quantitative prediction of soil Cr, there are many possibly acceptable models that may suit for in-situ application. When confronting other analytical targets, the methodology is still necessary to re-establish models for the different sample set. This can be the limitation of the studies on algorithms and methodologies. This research strengthened the stability and prediction accuracy of the binary modified DE algorithm combined with the parametric scaling SVM model, providing reference in support for future prediction of Cr and other heavy metal contents in large-scale soil contaminated areas. For different detection objects, it can be easily extended to train the models with different sample sets. Therefore, this study aims to verify the proposed modeling and calculation methodology would have great application potential.

## Supporting information

S1 File Raw data. (XLS)

S2 File Codes for PSSVM. (PDF)

S3 File Codes for BDE-PSSVM. (PDF)
