# Supplementary material for: Application of FT-NIR spectroscopy to the prediction of Chromium contamination in soil by evolutionary chemometrics
Source: PLoS One. 2026 Jan 27;21(1):e0341152. doi: 10.1371/journal.pone.0341152 (PMC12843573; doi:10.1371/journal.pone.0341152)
Supplement: S2 File — (PDF) [file pone.0341152.s002.pdf]

Codes for PSSVM

main.m

```
[train_x,train_y] = loadtraindata();
P_number=20;
C1=2;
C2=2;
W_max=0.95;
W_min=0.4;
K=100;
gammax=300;
gammin=0.01;
sig2max=200;
sig2min=0.01;
V_max=25;
V_min=-V_max;
P_xy=zeros(P_number,3);
P_v=zeros(P_number,2);
Pbest=zeros(P_number,3);
gbest=zeros(1,3);

for i=1:P_number
    Pbest(i,1)=(gammax-gammin)*rand+gammin;
    P_xy(i,1)=Pbest(i,1);
    Pbest(i,2)=(sig2max-sig2min)*rand+sig2min;
    P_xy(i,2)=Pbest(i,2);
    Pbest(i,3)=adapt(Pbest(i,1),Pbest(i,2),train_x,train_y);
    P_xy(i,3)=Pbest(i,3);
    P_v(i,1)=V_max*rands(1,1);
    P_v(i,2)=V_max*rands(1,1);
end

gbest(1,1)=(gammax-gammin)*rand+gammin;
gbest(1,2)=(sig2max-sig2min)*rand+sig2min;
gbest(1,3)=adapt(gbest(1,1),gbest(1,2),train_x,train_y);

for j=1:K
    for i=1:P_number
        i=1;
        gam=P_xy(i,1);
        sig2=P_xy(i,2);
        ad=adapt(gam,sig2,train_x,train_y);
        P_xy(i,3)=ad;
        if ad<Pbest(i,3)
```

```
                Pbest(i,1)=gam;
                Pbest(i,2)=sig2;
                Pbest(i,3)=ad;
            end
            if Pbest(i,3)<gbest(1,3)
                gbest(1,1)=Pbest(i,1);
                gbest(1,2)=Pbest(i,2);
                gbest(1,3)=Pbest(i,3);
            end

            W=W_min+(W_max-W_min)*(1-j/K);
            P_v(i,1)=W*P_v(i,1)+C1*rand*(Pbest(i,1)-P_xy(i,1))+C2*rand*(gbest(1,1)-
P_xy(i,1));
            if(P_v(i,1)>V_max)
                P_v(i,1)=V_max;
            end
            if(P_v(i,1)<V_min)
                P_v(i,1)=V_min;
            end
            P_v(i,2)=W*P_v(i,2)+C1*rand*(Pbest(i,2)-P_xy(i,2))+C2*rand*(gbest(1,2)-
P_xy(i,2));
            if(P_v(i,2)>V_max)
                P_v(i,2)=V_max;
            end
            if(P_v(i,2)<V_min)
                P_v(i,2)=V_min;
            end
            P_xy(i,1)=P_xy(i,1)+P_v(i,1);
            if(P_xy(i,1)>gammax)
                P_xy(i,1)=gammax;
            end
            if(P_xy(i,1)<gammin)
                P_xy(i,1)=gammin;
            end
            P_xy(i,2)=P_xy(i,2)+P_v(i,2);
            if(P_xy(i,2)>sig2max)
                P_xy(i,2)=sig2max;
            end
            if(P_xy(i,2)<sig2min)
                P_xy(i,2)=sig2min;
            end
        end
end
```

adapt.m
function ad=adapt(gam,sig2,train_x,train_y)<br>ad=rclssvm(gam,sig2,train_x,train_y);<br>end

loadtraindata
function [train_x,train_y]=loadtraindata()<br>train_x=importdata('C:\Users\admin\Desktop\pso-lssvm\1_data\train_x.mat');<br>train_y=importdata('C:\Users\admin\Desktop\pso-lssvm\1_data\train_ctly.mat');<br>end

lssvm1.m

```
clc
clear all
data1=xlsread('spxy.xlsx','A1:BGX79');
data2=xlsread('spxy.xlsx','A1:BGX79');

xx=data1(:,2:end);
train_y= data1(:,1);

[n1,m1]=size(xx);
mu1=mean(xx,2);
e1=xx-repmat(mu1,1,m1);
train_x=e1./repmat(sqrt(sum(e1.^2,2)/(m1-1)),1,m1);

yy=data2(:,2:end);
test_y=data2(:,1);

[n2,m2]=size(yy);
mu2=mean(yy,2);
e2=yy-repmat(mu2,1,m2);
test_x=e2./repmat(sqrt(sum(e2.^2,2)/(m2-1)),1,m2);

type='f';
kernel='RBF_kernel';
preprocess='original';
gam=1;
sig2=2;
model=initlssvm(train_x,train_y,type,gam,sig2,kernel,'original')
model=trainlssvm(model);
prey=simlssvm(model,test_x);
```

```
j1=size(prey,1);
d1=mean(prey);
aa1=prey-repmat(d1,j1,1);
s1=sum(aa1.^2);
rsd1=(sqrt(s1/(j1-1))/d1)*100;

g1=test_y-prey;
df1=sum(g1.^2);
rmse1=sqrt(df1/(j1-1));

sd1=sqrt(s1/(j1-1));
rpd1=(sd1/rmse1);

R1=corr(test_y,prey);

qd=[rsd1,rmse1,rpd1,R1];
```

predic1.m

```
function [rmse1]=fobj1(input_train,output_train,type1,cv1,cv2,kernel1,preprocess1)
model=initlssvm(input_train,output_train,type1,cv1,cv2,kernel1,preprocess1)
model=trainlssvm(model);
prey=simlssvm(model,input_train);

j1=size(prey,1);
d1=mean(prey);
g1=output_train-prey;
df1=sum(g1.^2);
rmse1=sqrt(df1/(j1-1));
```

rclssvm.m

```
function error=rclssvm(gam,sig2,train_x,train_y)
model= trainlssvm({train_x,train_y,'f',gam,sig2,'RBF_kernel'});
y=simlssvm(model,train_x);
error=sqrt(sum((train_y-y).^2)/length(train_y));
end
```

train1.m

```
clc
```

```
clear all
n1=1:2:200;
x1=sin(n1*0.1);
n2=2:2:200;
x2=sin(n2*0.1);
xn_train=n1;
dn_train=x1;
xn_test=n2;
dn_test=x2;
X=xn_train';
Y=dn_train';
Xt=xn_test';
Yt=dn_test';
type='f';
kernel='RBF_kernel';
gam=100;
sig2=0.01;
model=initlssvm(X,Y,type,gam,sig2,kernel);
costfun='crossvalidate';
costfun_args={X,Y,10};
optfun='gridsearch';
model=tunelssvm(model,[],optfun,{},costfun,costfun_args);

model=trainlssvm(model);
Yd=simlssvm(model,Xt);
```
