# Supplementary material for: Application of FT-NIR spectroscopy to the prediction of Chromium contamination in soil by evolutionary chemometrics
Source: PLoS One. 2026 Jan 27;21(1):e0341152. doi: 10.1371/journal.pone.0341152 (PMC12843573; doi:10.1371/journal.pone.0341152)
Supplement: S3 File — (PDF) [file pone.0341152.s003.pdf]

Codes for BDE-PSSVM

lin_DElssvm.m

```
clc
clear all
data1=xlsread('spxy1.xlsx','A1:BGX79');
data2=xlsread('spxy2.xlsx','A1:BGX79');

xx=data1(:,2:end);
train_y= data1(:,1);

[n1,m1]=size(xx);
mu1=mean(xx,2);
e1=xx-repmat(mu1,1,m1);
train_x=e1./repmat(sqrt(sum(e1.^2,2)/(m1-1)),1,m1);

yy=data2(:,2:end);
test_y=data2(:,1);

[n2,m2]=size(yy);
mu2=mean(yy,2);
e2=yy-repmat(mu2,1,m2);
test_x=e2./repmat(sqrt(sum(e2.^2,2)/(m2-1)),1,m2);


N=34;
gm=100;
CR=0.2;
bestc=0;
bestg=0;
error=inf;
Fmin=0.2;
Fmax=0.8;
dim=1;
lb=[2^-2];
ub=[2^8];
VarSize=[1,dim];
type='f';
kernel='lin_kernel';
preprocess='original';

kk=5;
gg=1;
```

```
cg=-2:0.3:8;
h12=2.^cg;
h121=h12';
Positions=h121;

BestSol.Cost=inf;
individual.Position=[];
individual.Cost=[];
BestSol.Cost=inf;
pop=repmat(individual,N,1);



pop1=zeros(N,1);
indices=crossvalind('Kfold',n1,kk);
    for k1=1:kkk=1010
   for i1=1:N
        test = (indices == k1);
        train = ~test;
        train_data=train_x(train,:);
        train_target=train_y(train,:);
        test_data=train_x(test,:);
        test_target=train_y(test,:);
        pop(i1).Position=Positions(i1,:);
         t=h12;

[fit1(k1,i1),fit2(k1,i1),R11(k1,i1)]=fobj5(train_data,train_target,test_data,test_target,type,t(1),
kernel,preprocess);
   end
    end
     for i4=1:N
  pop(i4).Cost =(sum(fit2(:,i4))/5)';
    end

BestCost=zeros(gm,1);
bestPosition1=zeros(gm,1);
bestPosition2=zeros(gm,1);

for k2=1:gm
    for i2=1:N
        p=pop(i2).Position;
        A=randperm(N);
        A(A==i2)=[];
  for i3=1:N-1
        popnew(i3).Position=pop(A(i3)).Position;
```

```
         t=popnew(i3).Position;
         popnew(i3).Cost=fobj6(train_x,train_y,type,t(1),kernel,preprocess);
         tt(i3)=popnew(i3).Cost;
end
        [row]=find(tt==min(tt));
        xbest=popnew(row(1)).Position;
        A(A==row(1))=[];
         a=A(1);
         b=A(2);
         c=A(3);
         d=A(4);

         F=unifrnd(Fmin,Fmax,VarSize);
         h=xbest+F.*(pop(a).Position-pop(b).Position);

          for j=1:dim
                h(j)=max(h(j),lb(j));
                  h(j)=min(h(j),ub(j));
             if j>1
                    h(j)=round(h(j))    ;
             else
                h(j)=h(j);
             end
          end

         z1=zeros(size(p));
         j0=randi([1,numel(p)]);
         for jd=1:numel(p)
             if jd==j0 || rand<=CR
                   z1(jd)=h(jd);
             else
                   z1(jd)=p(jd);
             end
         end
      NewSol.Position=z1;

      for k4=1:kk
   for i7=1:N
         test = (indices == k1);
         train = ~test;
         train_data=train_x(train,:);
         train_target=train_y(train,:);
         test_data=train_x(test,:);
         test_target=train_y(test,:);
```

```
[fit5(k4,i7),fit6(k4,i7),R13(k4,i7)]=fobj5(train_data,train_target,test_data,test_target,type,NewSol.Position(1),kernel,preprocess);
     end
         end
  NewSol.Cost = sum((sum(fit6)/5) /34);

              if NewSol.Cost<pop(i2).Cost
                      pop(i2)=NewSol;
                      if pop(i2).Cost<BestSol.Cost
                            BestSol=pop(i2);
                      end
              end
       end

       BestCost(k2)=BestSol.Cost;
       bestPosition1(k2)=BestSol.Position(1,1);
end

best=[bestPosition1,BestCost];
bestc=BestSol.Position(1,1);
bestg=0;

model=initlssvm(train_x,train_y,type,bestc,[],kernel,preprocess);
model=trainlssvm(model);
prey1=simlssvm(model,train_x);
prey2=simlssvm(model,test_x);
result1=[train_y prey1];
result2=[test_y prey2];

j1=size(prey1,1);
d1=mean(prey1);
aa1=prey1-repmat(d1,j1,1);
s1=sum(aa1.^2);
rsd1=(sqrt(s1/(j1-1))/d1)*100;

j2=size(prey2,1);
d2=mean(prey2);
aa2=prey2-repmat(d2,j2,1);
s2=sum(aa2.^2);
rsd2=(sqrt(s2/(j2-1))/d2)*100;

g1=train_y-prey1;
df1=sum(g1.^2);
rmse1=sqrt(df1/(j1-1));
```

```
g2=test_y-prey2;
df2=sum(g2.^2);
rmse2=sqrt(df2/(j2-1));

sd1=sqrt(s1/(j1-1));
rpd1=(sd1/rmse1);

sd2=sqrt(s2/(j2-1));
rpd2=(sd2/rmse2);

R1=corr(train_y,prey1);
R2=corr(test_y,prey2);
qd=[rsd1,rmse1,rpd1,R1,bestc;rsd2,rmse2,rpd2,R2,bestg]';
```

poly_DElssvm.m

```
clc
clear all
data1=xlsread('spxy1.xlsx','A1:BGX79');
data2=xlsread('spxy2.xlsx','A1:BGX79');

xx=data1(:,2:end);
train_y= data1(:,1);

[n1,m1]=size(xx);
mu1=mean(xx,2);
e1=xx-repmat(mu1,1,m1);
train_x=e1./repmat(sqrt(sum(e1.^2,2)/(m1-1)),1,m1);

yy=data2(:,2:end);
test_y=data2(:,1);

[n2,m2]=size(yy);
mu2=mean(yy,2);
e2=yy-repmat(mu2,1,m2);
test_x=e2./repmat(sqrt(sum(e2.^2,2)/(m2-1)),1,m2);

N=34;
gm=100;
CR=0.2;
bestc=0;
bestg=0;
error=inf;
```

```
Fmin=0.2;
Fmax=0.8;
dim=2;
lb=[2^-2,2];
ub=[2^8,6];
VarSize=[1,dim];
type='f';
kernel='poly_kernel';
preprocess='original';

kk=5;
gg=1;

cg1=-2:0.3:8;
cg2=2:1:6;
[n3,m]=size(cg1);
n=numel(cg1);
as1=2.^(cg1);
as21=[repmat(cg2,1,7)];
as2=as21(:,1:34);
h12=[as1;as2]';
Positions=h12;

BestSol.Cost=inf;
individual.Position=[];
individual.Cost=[];
BestSol.Cost=inf;
pop=repmat(individual,m,1);

pop1=zeros(N,1);
indices=crossvalind('Kfold',n1,kk);
    for k1=1:kk
   for i1=1:n
        test = (indices == k1);
        train = ~test;
        train_data=train_x(train,:);
        train_target=train_y(train,:);
        test_data=train_x(test,:);
        test_target=train_y(test,:);
        pop(i1).Position=Positions(i1,:);
         t=h12;

[fit1(k1,i1),fit2(k1,i1),R11(k1,i1)]=fobj2(train_data,train_target,test_data,test_target,type,t(1),
t(2),gg,kernel,preprocess);
```

```
    end
      end
        for i4=1:N
  pop(i4).Cost =(sum(fit2(:,i4))/5)';
      end

BestCost=zeros(gm,1);
bestPosition1=zeros(gm,1);
bestPosition2=zeros(gm,1);

for k2=1:gm
      for i2=1:m
              p=pop(i2).Position;
              A=randperm(m);
              A(A==i2)=[];
  for i3=1:m-1
              popnew(i3).Position=pop(A(i3)).Position;
              t=popnew(i3).Position;
              popnew(i3).Cost=fobj4(train_x,train_y,type,t(1),t(2),gg,kernel,preprocess);
              tt(i3)=popnew(i3).Cost;
  end
            [row]=find(tt==min(tt));
            xbest=popnew(row(1)).Position;
            A(A==row(1))=[];
              a=A(1);
              b=A(2);
              c=A(3);
              d=A(4);

              F=unifrnd(Fmin,Fmax,VarSize);
              h=xbest+F.*(pop(a).Position-pop(b).Position);

                for j=1:dim
                        h(j)=max(h(j),lb(j));
                          h(j)=min(h(j),ub(j));
                      if j>1
                              h(j)=round(h(j))    ;
                      else
                          h(j)=h(j);
                      end
                end

              z1=zeros(size(p));
              j0=randi([1,numel(p)]);
```

```
            for jd=1:numel(p)
                  if jd==j0 || rand<=CR
                        z1(jd)=h(jd);
                  else
                        z1(jd)=p(jd);
                  end
            end
        NewSol.Position=z1;

        for k4=1:kk
     for i7=1:m
            test = (indices == k1);
            train = ~test;
            train_data=train_x(train,:);
            train_target=train_y(train,:);
            test_data=train_x(test,:);
            test_target=train_y(test,:);
[fit5(k4,i7),fit6(k4,i7),R13(k4,i7)]=fobj2(train_data,train_target,test_data,test_target,type,NewSol.Position(1),NewSol.Position(2),gg,kernel,preprocess);
     end
        end
  NewSol.Cost = sum((sum(fit6)/5) /34);

            if NewSol.Cost<pop(i2).Cost
                  pop(i2)=NewSol;
                  if pop(i2).Cost<BestSol.Cost
                      BestSol=pop(i2);
                  end
            end
      end

      BestCost(k2)=BestSol.Cost;
      bestPosition1(k2)=BestSol.Position(1,1);
      bestPosition2(k2)=BestSol.Position(1,2);

end

best=[bestPosition1,bestPosition2,BestCost];

bestc=BestSol.Position(1,1);
bestg=BestSol.Position(1,2);

model=initlssvm(train_x,train_y,type,bestc,[gg,bestg],kernel,preprocess);
model=trainlssvm(model);
```

```
prey1=simlssvm(model,train_x);
prey2=simlssvm(model,test_x);
result1=[train_y prey1];
result2=[test_y prey2];

j1=size(prey1,1);
d1=mean(prey1);
aa1=prey1-repmat(d1,j1,1);
s1=sum(aa1.^2);
rsd1=(sqrt(s1/(j1-1))/d1)*100;

j2=size(prey2,1);
d2=mean(prey2);
aa2=prey2-repmat(d2,j2,1);
s2=sum(aa2.^2);
rsd2=(sqrt(s2/(j2-1))/d2)*100;

g1=train_y-prey1;
df1=sum(g1.^2);
rmse1=sqrt(df1/(j1-1));

g2=test_y-prey2;
df2=sum(g2.^2);
rmse2=sqrt(df2/(j2-1));

sd1=sqrt(s1/(j1-1));
rpd1=(sd1/rmse1);

sd2=sqrt(s2/(j2-1));
rpd2=(sd2/rmse2);

R1=corr(train_y,prey1);
R2=corr(test_y,prey2);
qd=[rsd1,rmse1,rpd1,R1,bestc;rsd2,rmse2,rpd2,R2,bestg]';
```

RBF_DElssvm.m

```
clc
clear all
data1=xlsread('spxy1.xlsx','A1:BGX79');
data2=xlsread('spxy2.xlsx','A1:BGX79');

xx=data1(:,2:end);
train_y= data1(:,1);
```

```
  SNV
[n1,m1]=size(xx);
mu1=mean(xx,2);
e1=xx-repmat(mu1,1,m1);
train_x=e1./repmat(sqrt(sum(e1.^2,2)/(m1-1)),1,m1);
  3. 20
yy=data2(:,2:end);
test_y=data2(:,1);
  SNV
[n2,m2]=size(yy);
mu2=mean(yy,2);
e2=yy-repmat(mu2,1,m2);
test_x=e2./repmat(sqrt(sum(e2.^2,2)/(m2-1)),1,m2);

  DE
N=34;
gm=100;
CR=0.2;
bestc=0;
bestg=0;
error=inf;
Fmin=0.2;
Fmax=0.8;
dim=2;
lb=[2^-2,2^-4];
ub=[2^8,2^6];
VarSize=[1,dim];
type='f';
kernel='RBF_kernel';
preprocess='original';
  preprocess='preprocess';
kk=5;

cg1=-2:0.3:8;
cg2=-4:0.3:6;
  [n3,m]=size(cg1);
n=numel(cg1);
for rw=1:n
  as(rw)=2^(cg1(rw));
  as1(rw)=2^(cg2(rw)) ;
  h12=[as;as1];
end
h121=h12';
Positions=h121;
```

```
BestSol.Cost=inf;
individual.Position=[];
individual.Cost=[];
BestSol.Cost=inf;
pop=repmat(individual,m,1);


pop1=zeros(N,1);
indices=crossvalind('Kfold',n1,kk);
    for k1=1:kkk=1010
   for i1=1:n
        test = (indices == k1); test
        train = ~test;traintest
        train_data=train_x(train,:);train
        train_target=train_y(train,:);
        test_data=train_x(test,:);test
        test_target=train_y(test,:);
        pop(i1).Position=Positions(i1,:);
         t=h12(:,i1)';

[fit1(k1,i1),fit2(k1,i1),R11(k1,i1)]=fobj1(train_data,train_target,test_data,test_target,type,t(1),
t(2),kernel,preprocess);
   end
    end
     for i4=1:N
  pop(i4).Cost =(sum(fit2(:,i4))/5)';
     end
BestCost=zeros(gm,1);
bestPosition1=zeros(gm,1);
bestPosition2=zeros(gm,1);
 DE
for k2=1:gm
    for i2=1:m
        p=pop(i2).Position;

        A=randperm(m);
        A(A==i2)=[];
  for i3=1:m-1
        popnew(i3).Position=pop(A(i3)).Position;
         t=popnew(i3).Position;
         popnew(i3).Cost=fobj3(train_x,train_y,type,t(1),t(2),kernel,preprocess);
         tt(i3)=popnew(i3).Cost;
  end
       [row]=find(tt==min(tt));
```

```
            xbest=popnew(row(1)).Position; cg
            A(A==row(1))=[];
             a=A(1);
             b=A(2);
             c=A(3);
             d=A(4);
               Mutation
             F=unifrnd(Fmin,Fmax,VarSize);
             h=xbest+F.*(pop(a).Position-pop(b).Position+pop(c).Position-pop(d).Position);
        h=xbest+F.*(pop(a).Position-pop(b).Position);

   h=pop(a).Position+F.*(pop(b).Position-pop(c).Position);

             for i6=1:dim
             h=max(h,lb(i6));
                   h=min(h,ub(i6));
             end
                Crossover
             z1=zeros(size(p));
             j0=randi([1,numel(p)]);
             for jd=1:numel(p)
                   if jd==j0 || rand<=CR
                         z1(jd)=h(jd);
                   else
                         z1(jd)=p(jd);
                   end
             end
         NewSol.Position=z1;

         for k4=1:kkk=1010
      for i7=1:m
             test = (indices == k1); test
             train = ~test;traintest
             train_data=train_x(train,:);train
             train_target=train_y(train,:);
             test_data=train_x(test,:);test
             test_target=train_y(test,:);
[fit5(k4,i7),fit6(k4,i7),R13(k4,i7)]=fobj1(train_data,train_target,test_data,test_target,type,Ne
wSol.Position(1),NewSol.Position(2),kernel,preprocess);
      end
         end
                NewSol.Cost = sum((sum(fit6)/5) /34);
            if NewSol.Cost<pop(i2).Cost
                   pop(i2)=NewSol;
```

```
                if pop(i2).Cost<BestSol.Cost
                    BestSol=pop(i2);
                end
            end
        end
    Update Best Cost
        BestCost(k2)=BestSol.Cost;
        bestPosition1(k2)=BestSol.Position(1,1);
        bestPosition2(k2)=BestSol.Position(1,2);

end

best=[bestPosition1,bestPosition2,BestCost];
  cg
bestc=BestSol.Position(1,1);
bestg=BestSol.Position(1,2);

  LSSVM
model=initlssvm(train_x,train_y,type,bestc,bestg,kernel,preprocess);
model=trainlssvm(model);
prey1=simlssvm(model,train_x);
prey2=simlssvm(model,test_x);
result1=[train_y prey1];
result2=[test_y prey2];

  RSD
j1=size(prey1,1);
d1=mean(prey1);
aa1=prey1-repmat(d1,j1,1);
s1=sum(aa1.^2);
rsd1=(sqrt(s1/(j1-1))/d1)*100;

j2=size(prey2,1);
d2=mean(prey2);
aa2=prey2-repmat(d2,j2,1);
s2=sum(aa2.^2);
rsd2=(sqrt(s2/(j2-1))/d2)*100;
    RMSE
g1=train_y-prey1;
df1=sum(g1.^2);
rmse1=sqrt(df1/(j1-1));

g2=test_y-prey2;
df2=sum(g2.^2);
```

```
rmse2=sqrt(df2/(j2-1));
    RPD
sd1=sqrt(s1/(j1-1));
rpd1=(sd1/rmse1);

sd2=sqrt(s2/(j2-1));
rpd2=(sd2/rmse2);
  R^2
R1=corr(train_y,prey1);
R2=corr(test_y,prey2);
qd=[rsd1,rmse1,rpd1,R1,bestc;rsd2,rmse2,rpd2,R2,bestg]';
```
